# A Scoping Review of Knowledge, Awareness, Perceptions, Attitudes, and Risky Behaviors of Sexually Transmitted Infections in Southeast Asia

**DOI:** 10.3390/healthcare11081093

**Published:** 2023-04-11

**Authors:** Vimala Balakrishnan, Kok Khuen Yong, Chiong Kian Tiong, Nicholas Jian Shen Ng, Zhao Ni

**Affiliations:** 1 Faculty of Computer Science and Information Technology, Universiti Malaya, Kuala Lumpur 50603, Malaysia; 2 Faculty of Medicine, Universiti Malaya, Kuala Lumpur 50603, Malaysia; 3 School of Nursing, Yale University, New Haven, CT 06520, USA

**Keywords:** sexually transmitted infections, Southeast Asia, awareness, perception, risky behavior, scoping review

## Abstract

This scoping review synthesizes literature to examine the extent of research focusing on knowledge, awareness, perceptions, attitudes, and risky behaviors related to sexually transmitted infections (STIs) in Southeast Asia (SEA). The PRISMA-Scoping approach was adopted targeting articles published from 2018 to 2022, sought from CINALH, PubMed, Web of Science and Scopus. A process of screening and elimination resulted in a total of 70 articles reviewed. Most of the studies were conducted in Indonesia, Thailand, Vietnam, and Malaysia, with the majority focusing on HIV/AIDS. In general, studies examining knowledge, awareness, and risky behaviors related to STIs in SEA reported low levels across various cohorts. However, evidence suggests that these issues are more prominent among individuals with low levels of education or low socioeconomic status, those living in rural areas or those working in the sex/industrial sectors. Engaging in unsafe sex and having multiple partners are the key examples for risky sexual behavior, while fear of being rejected/discriminated/stigmatized and lacking STI awareness were identified as social risky behaviors in SEA. Overall, cultural, societal, economic and gender inequality (male dominance) greatly impact people’s knowledge, awareness, perceptions, attitudes, and risky behaviors in SEA. Education is an important factor influencing healthy behavior; therefore, this scoping review calls for increased investment in educating vulnerable populations to prevent STIs, particularly in less-developed countries/regions of SEA.

## 1. Introduction

Sexually transmitted infections (STIs) are caused by bacteria, viruses or parasites that spread predominantly through sexual contact including vaginal, anal, and oral sex. However, some of these diseases are known to spread through non-sexual means, such as blood or from mother to child during pregnancy and childbirth [1,2,3]. According to the World Health Organization (WHO) [4], more than 1 million STIs are acquired daily worldwide, with an estimated 374 million new infections of chlamydia, gonorrhea, syphilis, and trichomoniasis in 2020.

The prevalence of STIs is higher in low- and middle-income countries (LMIC), particularly among countries in the Southeast Asia (SEA) region. For instance, Thailand had an estimated 500,000 people living with HIV, with 12,000 people dying of AIDS-related causes in 2020 [5], and in Indonesia and Laos, HIV prevalence among individuals aged 15–49 years was reported to be growing from 2002 to 2021 at annual average rates of 8.33% and 7.89%, respectively [6]. Intervention strategies are available, though these are often hampered by low levels of knowledge and awareness, high levels of risky behaviors adopted, and a widespread stigma around STIs. As such, improving knowledge, awareness, perception, attitude, and risky behaviors about STIs is a critical component in intervention, prevention, and control strategies globally. However, there is lack of evidence in knowledge, awareness, perception, attitude, and risky behaviors among developing countries, particularly in the SEA region where STI prevalence is deemed to be high.

This scoping review aims to map the existing literature on knowledge, awareness, perceptions, attitudes, and risky behaviors related to STIs in SEA. Specifically, we aim to explore the levels of knowledge, awareness, perception, attitude, and risky behaviors, identifying the top risky behaviors, and examine the key sociodemographic factors that influence these factors across all cohorts based on publications from 2018 to 2022.

## 2. Materials and Method

The scoping review is part of a large review process targeting studies that have been conducted in the Asian region, focusing on knowledge, awareness, perception, attitude, and risky behaviors spanning from 2018 to 2022. A description of the method is provided below. The review follows the guidelines given in the PRISMA Extension for Scoping Reviews [7].

### 2.1. Research Questions

Four research questions (RQs) were formulated to guide this scoping review, as given below:

RQ1—What is the current landscape of studies reporting on knowledge, awareness, perception, attitude, and risky behaviors related to STIs in SEA?

RQ2—What are the levels of knowledge, awareness, attitude, and perception of STIs in SEA?

RQ3—What are the key sociodemographic profiles linked to knowledge, awareness, perception, attitude, and risky behaviors related to STIs?

RQ4—What are the top risky behaviors associated with STIs?

### 2.2. Search Strategy

Articles published from 2018 to 2022 were downloaded from four academic databases, namely PubMed, Web of Science (WoS), Scopus, and Cumulated Index to Nursing and Allied Health Literature (CINALH). The articles were limited to studies conducted within the Asian region. The main search strings are given below:

- (sex* transm* OR STI OR STD) AND (know* OR beh* OR aware* OR attitude* OR perce* OR stigma* OR risk*)
- (HIV OR HPV) AND (know* OR beh* OR aware* OR attitude* OR perce* OR stigma* OR risk*)

### 2.3. Study Selection

A total of 31,021 articles were identified based on the search strings above, and these were then filtered using the inclusion and exclusion criteria. Table 1 shows the specific criteria used for this scoping review, which resulted in the removal of 28,886 articles.

**Figure 1:**
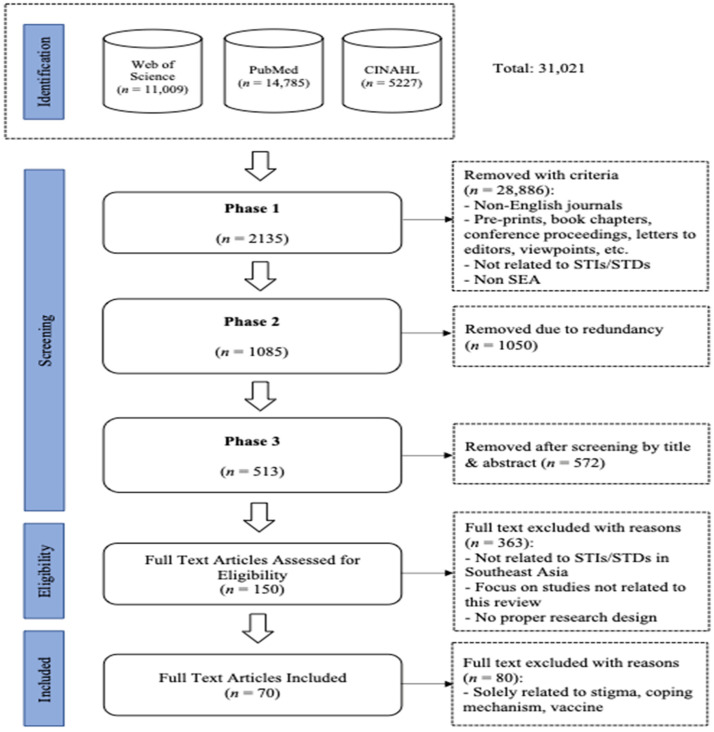
PRISMA-ScR flowchart.

1050 duplicate entries were removed, resulting in 1085 articles. These were then examined for their appropriateness based on the titles and abstracts by three reviewers. Disagreements (if any) were resolved by the fourth reviewer. This step resulted in 513 articles. A further eligibility check was performed whereby all 513 articles were checked in entirety to ensure all the criteria in Table 1 were fulfilled. The final number of articles deemed suitable for this review was 70. The PRISMA-Scoping diagram outlining these steps are given in Figure 1.

### 2.4. Charting the Data

At this stage, all 70 articles were reviewed and relevant data addressing the four RQs in Section 2.1 were extracted. Specifically, details extracted include author names and year of publication, country of study, aim, cohort, focus (i.e., specific elements of knowledge, awareness, perception, attitude, and risky behaviors), instruments, and key results etc. This was accomplished by three reviewers, and the results were cross-checked by the fourth reviewer to minimize errors. The data extraction was done using Microsoft Excel.

### 2.5. Collating, Summarizing, and Reporting the Results

In this final stage, the outcomes of the review are summarized and presented using tables and charts as deemed fit. These are presented in the following section based on the RQs.

## 3. Results and Discussion

### 3.1. Study Characteristics

This section answers RQ1 through focusing on the current landscape of studies reporting on knowledge, awareness, perception, attitude, and risky behaviors and STIs in SEA. Table 2 shows the descriptive statistics of the studies reviewed, with the number of studies published ranging from 7 (2022) to 20 (2019). Indonesia (*n* = 24), Thailand (*n* = 21), Vietnam (*n* = 17), and Malaysia (*n* = 10) emerged to be the top four countries followed by Cambodia (*n* = 5). Philippines [1,8] and Singapore [8,9] had two studies each whilst only a single study was found for Myanmar and Laos [8]. No studies were found in Brunei and Timor Leste. The majority of the studies focused on HIV/AIDS (67.1%), followed by Human Papilloma Virus (HPV) (19%) and other STIs (13.9%).

The top three points of focus among knowledge, awareness, perception, attitude, and risky behaviors include knowledge (*n* = 43), attitude (*n* = 20), and risky behavior (*n* = 17). Five focused on perception [1,10,11,12,13], and only two on awareness [14,15]. Questionnaire surveys (*n* = 40) and interviews (*n* = 29) were the two most popular methods used in data collection. Three studies relied on existing datasets [16,17,18], while two more used the focus group method [11,12].

Most of the studies focused on students, namely those in high school (*n* = 10) and university (*n* = 9), followed by high-risk populations such as prisoners, migrants and sex workers (*n* = 9). Six studies were found to have targeted healthcare workers (HCWs) and (young) men having sex with men (MSM), and seven studies targeted women in general. Finally, the most common sociodemographic/behavioral factors in studies were education level (*n* = 28), age (*n* = 21), income level (*n* = 19), marital status (*n* = 11), sex (*n* = 9), and occupation (*n* = 9). Sexual behavioral risk, ethnicity, and place of birth each had five studies, while religion [19,20,21], underlying conditions [22,23,24], and an infected living partner had three studies each [22,25,26].

### 3.2. Levels of Knowledge, Awareness, Attitude, and Perception in SEA

To answer RQ2, we extracted the relevant data from studies that have specifically reported these figures. Table 3 provides a summary based on the topic of investigation and cohorts. A general observation shows a low level of knowledge among the studies, regardless of the cohorts and type of STIs. For example, knowledge levels for HPV were low not only among rural women [1], but university students as well [14]. Interestingly, the latter found Malaysian university students were only moderately aware, as 59.8% have heard about HPV (*n* = 425), with women having a marginally higher knowledge than men. An almost equal split of knowledge level was observed in Indonesia among 400 urbanites (50.8%), although a higher percentage (82%) reported a positive attitude towards regular HPV screenings.

It is noteworthy that high levels of knowledge about HIV/AIDS have been reported among high school students (96%) [31] and women (88.7%) [33]. For example, Rahman and his colleagues found that most of their participants had obtained knowledge about HIV/AIDS from mass media, evidenced by the high percentage of the use of television in providing such information. As for [33], the high knowledge level was mainly due to women’s demographic profiles, whereby most had high levels of education, wealth index and exposure to media/information/knowledge etc. Interestingly, the authors found that despite having a high HIV/AIDS knowledge, there is an overall negative attitude towards people living with HIV/AIDS (PLWHA) (i.e., 60.28%), thus showing that a high proportion of people with knowledge does not translate to more positive attitudes. Contrarily, another Indonesian study among 209 dentists found that only about 44% had good knowledge of HIV/AIDS, though slightly more than half the sample (53%) had a positive attitude towards PLWHA [32]. The authors agreed that the attitude score is deemed low (negative) considering that the cohort under-study is HCW, and thus they should be less discriminatory in treating PLWHA. In conclusion, findings show more education and awareness are needed to increase individuals’ knowledge, awareness, attitude, and perception to eliminate HIV-related stigma and discrimination in SEA.

In a study conducted among 60 female inmates of women shelters in Malaysia, the findings were mixed. While only 33% of the participants had good knowledge of STIs, they had an average score of 23.1 out of 25 for attitude towards STIs [18], indicating that most participants agreed they would oppose premarital sex and multiple sex partners, and would seek medical help if diagnosed with STIs. The authors generally found that many of the females opposed pre-marital sex (including people with STIs) and multiple sex partners, as well as acceptance of treatment if one is infected. Surprisingly, despite a negative attitude toward having multiple sex partners, almost half of the sample (*n* = 43) reported to have had more than one partner in the last three years. Such findings indicate that having sufficient knowledge alone does not necessarily guarantee an individual’s safe sexual practice.

### 3.3. Sociodemographic Profiles

Table 4 presents the summary of the sociodemographic profiles affecting the levels of knowledge, awareness, attitude, or perception related to STIs, as per RQ 3. It is noted that a study is listed in Table 4 if it reports at least one significant result (*p* < 0.05) for knowledge, awareness, attitude, or perception. Categorization as high or low is determined by the respective studies.

Based on the sex profile, most of the studies reported females to have higher levels of knowledge, awareness, attitude, and perception compared to males (*n* = 7). It is to noted however that a fair comparison is not possible, as many of these studies had a skewed distribution whereby females outnumbered males [10,40,43]. Additionally, some of these studies also specifically targeted female participants [1,14,39]. Nevertheless, some of the reasons provided by researchers for a lower level of knowledge and awareness among males include having a more uninvolved parenting style or lower parental monitoring, as well as more sex approval (for younger males/boys), and thus there is a lack of responsible communication on STIs among this cohort [40].

Most of the studies also show higher levels of knowledge, awareness, attitude, and perception among those with a higher education (*n* = 12), akin with higher socioeconomic status [2,9]. It is claimed that individuals with a higher education level (regardless of their cohorts) are better in accurately reading, processing, and understanding written and verbal information from sources such as newspapers, magazines, health pamphlets etc. Furthermore, such individuals are more exposed to social media and are more adept in seeking information related to STIs, unlike those with a lower education level. Being in a higher socioeconomic stratum also indicates that such individuals have means to own/access media such as technology devices (smartphones, computer, tablets), Internet access and/or televisions, and thus have better access to educational programmes pertaining to STIs [1,2,35].

Higher education and socioeconomic status can be linked to living area as well, with the majority of studies reporting a higher level of knowledge, awareness, attitude, and perception among urbanites [2,27] as opposed to those living in rural areas [39]. Individuals living in urban areas are known to have better access to information through the Internet, healthcare centers, NGOs etc., a notion supported by other studies reporting individuals with better accessibility to medical device and information as having higher levels of knowledge, awareness, attitude, and perception [25,44,46,48,49] than those without [39,42].

In terms of religiosity, studies involving participants from conservative nations such as Malaysia [20] or Muslim/Christian participants reported low levels of knowledge, awareness, attitude, and perception. For example, university students in Malaysia reported the absence of a comprehensive education of sex, relationships, and STIs, with teachers preaching abstinence. In fact, openly discussing the practicalities of safe sex and condom use, for example, is deemed as taboo in the country [20]. Being a Christian woman was found to have a significant association with a low level of HPV knowledge [41], most likely as staunch Catholics abstain from pre-marital sex, and thus do not seek information pertaining to said disease. In contrast, a study conducted in Laos reported that participants who followed any religion that accepts the use of birth control had higher levels of knowledge and awareness regarding STIs [35].

Finally, results based on participants’ occupation status revealed healthcare professionals, including medical students, to have a higher level of knowledge, awareness, attitude, and perception—a pattern that is absolutely expected considering their background [11,14,17,32]. Contrarily, the knowledge level was found to be extremely low among professions such as industrial workers (migrants) [42] and sex workers [29,50]. For example, [42] found a low average score for STI knowledge among 289 industrial workers in Vietnam. Similarly, [29] found a very low level of STI knowledge among male sex workers, especially among those who started serving clients at a young age, and therefore have not been exposed to safe sex practices. Overall, research suggests that lower levels of knowledge, awareness, attitude, and perception regarding STIs tend to be among socioeconomically disadvantaged populations, particularly in less developed countries/regions such as Vietnam [42] and Indonesia [29,50]. This is probably due to gaps in policies and healthcare access targeting these vulnerable cohorts, and thus the findings suggest the need for a higher investment in educational interventions to promote STI knowledge in this population. Providing stronger incentives for local community HCWs and NGOs to educate these individuals about STIs prevention and sexual health may also help promote higher knowledge.

### 3.4. Risky Behaviors and STIs

This final subsection addresses RQ4 by focusing on the risky behaviors related to STIs. We categorize these behaviors as sexual (e.g., multiple sexual partners and having unprotective sex) and social, for any behaviors that are not directly related to sexual activities such as discrimination or stigmatization [26,51]. Table 5 presented the social and sexual risky behaviors reported in studies conducted in SEA.

For risky sexual behaviors, the two most prominent behaviors include having unsafe sex (*n* = 15) and having multiple sexual partners (*n* = 12), both of which were mainly reported by studies targeting vulnerable/high-risk cohorts, including (Y)MSM and transgender people [26,53,54,59], female sex workers (FSW) and their clients [50,55,56], adolescents [13,40,51,52], and HIV-positive individuals [57,58]. For instance, a study involving 506 HIV individuals in Vietnam found that 83.2% of participants engage in sex without using a condom, and 27.9% had multiple sex partners. Studies specifically focusing on FSWs and their clients [50,55,56] show a very low negotiation in condom use—a behavior that was found to be significantly associated with HIV-risk sexual behavior. A low proportion of FSWs negotiating condom use in Indonesia [50,56] and Vietnam [55] reflects a cultural issue where male dominance determines the final decision on condom use or safe sex.

Interestingly, having unprotected sex and/or multiple sex partners were reported to be more common among male adolescents [40,52] and university students [8]. Such a tendency was particularly noted among boys with low parental control/monitoring [40] and male students who live away from their parents [8], thus suggesting that these individuals choose to engage in sexual risky behaviors due to a sense of freedom. Furthermore, this also provides an insight into a potentially risky environment in the community, particularly norms regarding males’ sexual behavior. An interesting point to note is that despite being young, the dominance of men in influencing condom use is profound in SEA. Unprotected sex and having multiple sex partners are often reported as root causes of STIs, and therefore urgent interventions, including at the school level, are necessary.

In the context of risky sexual behaviors, fear of being rejected/stigmatized/discriminated against (*n* = 4) and lacking knowledge and awareness emerged as the top two most-reported behaviors (*n* = 9). Lack of awareness/knowledge about STIs was not limited to high-risk or vulnerable cohorts, though they were also observed among youths and students [20,49]. For instance, Malaysian university students reported a lack or absence of sex education, particularly during their teenage years, with the majority relying on the Internet or teachers who informally preach abstinence without any discussion of the practicalities of condom use and safe sex. A similar observation was made by [49], based on a large-scale study involving 22,864 youths in Indonesia, with results indicating a mere 14.1% having knowledge of HIV. Similar to the Malaysian participants, more than half the sample relied on peers or oneself for sex-related discussions. One of the main reasons for this could be religion, with both countries being mostly populated by Muslims, and thus, tending to be more conservative. These findings further emphasize the importance of sex education being introduced to age-appropriate children, and implemented based on the sensitivity of cultural norms, local beliefs, and practices.

Fear of rejection/stigmatization/discrimination was also identified as a key risky social behavior for STIs. Individuals who are at a higher risk in contracting STIs were reported to be afraid to seek information or help from the society with matters pertaining to these diseases for fear of being stigmatized by society—a phenomenon particularly observed among cohorts such as FSW clients [56] and female migrant workers [19]. For example, female migrant workers (*n* = 18) in Thailand refrained from disclosing to having multiple sexual partners to their husbands/community for fear of marital conflict, resulting in them living in secrecy and engaging in unsafe sex (low condom use negotiation), with the latter also suggesting that male dominance over women influenced decision-making for safe sex [19]. A similar observation was also noted among university students in Malaysia where participants reported avoiding seeking STI-related information or going for free HIV testing (especially in government health centers) for fear of being discriminated or judged [20]. Such environments, including societal perception, undermine STI prevention, thus leading to increased levels of risky behavior.

Various other risky behaviors were reported, such as difficulty in purchasing condoms [20], substance/alcohol use [40,50,57], and partner/childhood sex abuse [23,52], among others. Other risky behaviors that are related to HIV transmission, such as injecting drugs [22,61] and sharing needles [37], were also reported. Interestingly, difficulty in purchasing condoms was reported in a study among Malaysian university students [20], with the participants mainly citing embarrassment associated with purchasing condoms as a barrier to their use in preventing HIV transmission, as buying condoms is often seen as a taboo—another pattern observed in a conservative nation. Substance/alcohol abuse, particularly consumption prior to engaging in sexual activities, was found to be associated with sexual and social risky behaviors [22,40,50,57]. For example, FSWs who had high levels of alcohol consumption (before or during sex) tend to engage in unsafe sex, as their abilities to negotiate safe sex (using condoms) are affected, along with the desire to use them [50]. Men and women who consume drugs such as heroin and crystal meth were also found to engage in high-sexual risk behaviors [22,23]. Findings generally show that sexual intercourse under the influence of drugs/alcohol reduces an individual’s self-control of sexual behaviors, leading to impulsive risky sexual behavior.

## 4. Conclusions

The scoping review examined the knowledge, awareness, perception, attitude, and risky behaviors related to STIs in SEA based on scientific papers published in the last five years. Most studies were conducted in Indonesia, Thailand, Vietnam, and Malaysia, and reported on various cohorts such as high-risk populations (i.e., MSMs, FSWs, migrant workers), adolescents, students, HCWs, and women. Low levels of knowledge and awareness, as well as negative perceptions and attitudes, were reported by the majority of the studies—a pattern that is particularly profound among individuals with low levels of education and low socioeconomic status, rural residents, and sex workers. Most high-risk populations were found to engage in unsafe sex practices, including having multiple partners, due to a low condom use negotiation skill, which were further exacerbated by alcohol/substance abuse, as well as a lack of awareness and education on STIs and safe sex practices. The review highlights the urgent need for targeted interventions to improve STI education, access to resources, and addressing substance abuse, which are important to mitigate the spread of STIs in SEA.

## Figures and Tables

**Table 1:** Inclusion and exclusion criteria.

Inclusion	Exclusion
Published articles, including those in press	Pre-prints, along with book chapters, conference proceedings, letters to editors, viewpoints etc.
Focus on knowledge, awareness, perception, attitude, or risky behavior (at least one)	Focus on stigmatization, prevalence, coping strategies and effect of intervention strategies on these aspects
Any STIs	Non-STIs such as cervical cancer, and vaccines
Valid research methodology (i.e., empirical, experiments, content analysis etc.)	Clinical biological trials that did not investigate participants’ knowledge, awareness, perceptions, or attitudes
Articles in English	
Location of study is Southeast Asia	

**Table 2:** Descriptive statistics.

Characteristics		*N* (%)
Year (*n* = 70)	2018	11 (15.7)
	2019	20 (28.6)
	2020	13 (18.6)
	2021	19 (27.1)
	2022	7 (10.0)
Country (*n* = 83) *	Vietnam	17 (20.5)
	Indonesia	24 (28.9)
	Thailand	21 (25.3)
	Malaysia	10 (12.0)
	Philippines	2 (0.02)
	Laos	1 (0.01)
	Cambodia	5 (0.06)
	Myanmar	1 (0.01)
	Singapore	2 (0.02)
Aspect (*n* = 79) *	HIV/AIDS	53 (67.1)
	HPV	11 (13.9)
	STD/STI	15 (19.0)
Focus (*n* = 87) *	Knowledge	43 (49.4)
	Risky behavior	17 (19.5)
	Attitude	20 (23.0)
	Awareness	2 (0.02)
	Perception	5 (0.06)
Methodology (*n* = 74) *	Survey	40 (54.1)
	Interview	29 (39.1)
	Dataset	3 (0.04)
	Focus group	2 (0.03)
Scale (*n* = 84) *	Own	44 (52.4)
	Database	4 (0.05)
	IDHS 2017	4 (0.05)
	IDHS 2012	2 (0.02)
	Others: Full list given at the end the table	16 (0.19)
Cohort (*n* = 72) *	University students	9 (12.5)
	Prisoners/migrants/sex workers	9 (12.5)
	General population	7 (0.10)
	HCW	6 (0.08)
	(Y)MSM/Trans.	6 (0.08)
	High-school students/adolescents	10 (13.9)
	Women	7 (0.10)
	Others: HIV patients, parents, teachers, people living with HIV patients etc.	18 (25.0)
Sociodemographic/behavioral (*n* = 125) *	Education level	28 (22.40)
	Age	21 (16.80)
	Income level	19 (15.20)
	Marital status	11 (8.80)
	Gender	9 (7.20)
	Occupation	9 (7.20)
	Social behavioral risk	6 (4.80)
	Sexual behavioral risk	5 (4.00)
	Ethnicity	5 (4.00)
	Place of birth	5 (4.00)
	Place of residence	4 (3.20)
	Religion	3 (2.40)
	Underlying conditions	3 (2.40)
	Infected living partner	3 (2.40)

Note: “*n*”—the frequency that an item was described in the included articles. *—Numbers do not total 70 due to multiple points of focus, countries etc. Others:—refer to instances with fewer than 3 studies; Only points of focus related to the review are shown. HIV—human immunodeficiency virus; HPV—human papillomavirus; AIDS—acquired immunodeficiency syndrome; STD—sexually transmitted disease; IHCW—Healthcare Worker; DHS—Indonesia Demographic Health and Survey; Scale for Others: (1) The Sensitivity to Punishment and Sensitivity to Reward Questionnaire; The Drug Abuse Screening Test; & The Sexual Risk Behavior; Vietnam Administrative of HIV/AIDS Control (VAAC); Safe Sexual Behavior Questionnaire; Condom Influence Strategy Questionnaire (CISQ); Asian Value Scale (AVS) and the Beliefs and Values Scales; Integrated Biological and Behavioral Surveillance (IBBS) 2012 questionnaire; Questionnaire based on guidelines from the Vietnam Ministry of Health and relevant references from the National Institute of Hygiene and Epidemiology; US NIH Pediatric HIV/AIDS Cohort Study Adolescent Master Protocol; National Youth Risk Behavior Survey questionnaire; Model Questionnaires of the DHS Program; EuroQol five-dimension five-level (EQ-5D-5L).

**Table 3:** Level of knowledge, awareness, attitude, or perception.

Topic	Study	Cohort, Size	Knowledge *		Awareness		Positive Attitude/Perception	
			Level (%)	Scale (Mean/Max)	Level (%)	Scale	Level (%)	Scale
HPV	[1]	Rural women, *n* = 338		6.40/15				
	[14]	University students, *n* = 425		5.26/13	59.8			
	[27]	Urbanites, *n* = 400	50.8				82.0	
HIV/AIDS	[28]	Highlanders/natives, *n* = 8039		3.10/6				
	[29]	MSW, *n* = 100	24.0					
	[16]	Housewives, *n* = 100	35.0					
	[30]	School students, *n* = 516		19.90/35				
	[31]	School students, *n* = 320	96.0					
	[32]	Dentists, 209	44.0				53.0	
	[33]	Women, *n* = 25,895	88.7				39.7	
	[34]	Industrial workers, *n* = 289		7.50/11				
	[17]	Pharmacy students, *n* = 1013		14.14/21				
		Pharmacists, *n* = 250		15.39/21				
	[15]	Adults, *n* = 403			14.0			
STD/STI	[35]	School students, *n* = 337	51.9					
	[36]	University students, *n* = 600		24.09/38				
	[37]	Muslim army conscripts, *n* = 360	21.3					
	[18]	Inmates of women shelter *n* = 60	33.0					23.1/25
	[38]	Boys, *n* = 10,547					29.8	

Note: Only studies that reported these figures are included in this table; Scales are not mentioned as most studies assessed using their own scales. *—Indicate possession of knowledge based on population (x/sample).

**Table 4:** Summary of sociodemographic profiles affecting the levels of knowledge, awareness, attitude, or perception.

Sociodemographic Profiles		Levels of Knowledge, Awareness, Attitude, or Perception	
		High	Low
		Studies	
Sex	Female	[10,14,27,39,40,41,42]	[1,43]
Education level	High	[1,2,9,27,28,33,34,35,44,45,46,47]	
	Low		[15,16,39,41,42]
Socioeconomic status	High	[2]	
	Low		[41]
Living area	Rural		[39]
	Urban	[2,27]	
Religion	Muslim/Christian		[20,41]
	Open	[35]	
Accessibility to medical device or information	High	[25,44,46,48,49]	
	Low		[39,42]
Occupation	Sex workers/Industrial		[29,42,50]
	Healthcare professionals/Medical students	[11,14,17,32]	

Note: Only sociodemographic profiles with more than two studies are included in this table; significant results are only as determined and reported in the respective studies (*p* < 0.05); a higher level of attitude/perception refers to more positive attitude/perception.

**Table 5:** Top STD/STI risky behaviors.

Type	Risky Behavior	Cohort	
Sexual	No/infrequent use of protective and/or preventive measures	Rural (women)	[1]
		Migrant workers	[3]
		Adolescents	[40,51,52]
		(Y)MSM/Transgender	[26,53,54]
		FSW and clients, inmates	[18,50,55,56]
		HIV patients	[57,58]
		University students	[8]
	Multiple sex partners	(Y)MSM/Transgender	[53,54,59]
		FSW and clients	[55,56]
		HIV patients	[57,58]
		University students	[8]
		Adolescents	[13,40,51]
		Adolescents (living with HIV)	[60]
		Married community	[12]
Social	Rejection, discrimination, and stigmatization	HIV (Female migrant workers)	[19]
		University students/youth	[20,49]
		FSW clients	[56]
	Lacking awareness and knowledge	(Y)MSM/Transgender	[26,53]
		Industry workers	[42]
		FSW and clients	[55,56]
		University students/Youth	[20,49]
		Adolescents	[51]
		Married community	[12]
Others	Injecting drugs, being sexually active, partner/childhood sex abuse, difficulty in condom procurement, substance/alcohol abuse, sharing needles		[20,22,23,37,40,50,52,55,57,61]

Only risky behaviors with a minimum of three studies were reported.

## Data Availability

No new data were created or analyzed in this study. Data sharing is not applicable to this article.
